# 4-chlorophenol removal from water using graphite and graphene oxides as photocatalysts

**DOI:** 10.1186/s40201-015-0184-0

**Published:** 2015-04-19

**Authors:** Karina Bustos-Ramírez, Carlos Eduardo Barrera-Díaz, Miguel De Icaza-Herrera, Ana Laura Martínez-Hernández, Reyna Natividad-Rangel, Carlos Velasco-Santos

**Affiliations:** Centro Conjunto de Investigación en Química Sustentable, UAEM-UNAM. Km.12 de la carretera Toluca-Atlacomulco, San Cayetano, 50200 Estado de México México; División de Estudios de Posgrado e Investigación, Instituto Tecnológico de Querétaro, Av. Tecnológico s/n Esq. M. Escobedo, Col. Centro Histórico, 76000 Querétaro, México; Centro de Física Aplicada y Tecnología Avanzada, Universidad Nacional Autónoma de México, Boulevard Juriquilla 3001, 76230 Querétaro, México

**Keywords:** 4-chlorophenol, Photocatalyst, Graphite oxide, Graphene oxide, Water pollution

## Abstract

Graphite and graphene oxides have been studied amply in the last decade, due to their diverse properties and possible applications. Recently, their functionality as photocatalytic materials in water splitting was reported. Research in these materials is increasing due to their band gap values around 1.8-4 eV, and therefore, these are comparable with other photocatalysts currently used in heterogeneous photocatalytic processes. Thus, this research reports the photocatalytic effectiveness of graphite oxide (GO) and graphene oxide (GEO) in the degradation of 4-chlorophenol (4-CP) in water. Under the conditions defined for this research, 92 and 97% of 4-CP were degraded with GO and GEO respectively, also 97% of total organic carbon was removed. In addition, by-products of 4-CP that produce a yellow solution obtained only using photolysis are eliminated by photocatalyst process with GO and GEO. The degradation of 4-CP was monitored by UV-Vis spectroscopy, High Performance Liquid Chromatography (HPLC) and Chemical Oxygen Demand (COD). Thus, photocatalytic activity to remove 4-CP from water employing GO and GEO without doping is successfully showed, and therefore, a new gate in research for these materials is opened.

## Background

With increasing global air and water pollutions, photocatalysis has attracted considerable attention because this method provides a promising pathway to attenuate environmental pollution problems, mainly due to the capacity of photocatalyst to degrade organic contaminants [1].

Chlorophenols represent an important group of organic water pollutants due to their toxicity and low biodegradability. They are considered priority pollutants and are employed in numerous industrial processes; in particular, 4-CP is involved in the synthesis of many pesticides, pharmaceuticals and dyes [2-5].

Most polluting organic compounds, including 4-CP, are difficult to degrade, so, it is important to develop more effective methods to promote their degradation [6]. Different biological, physical and chemical methods have been applied for chlorophenol degradation [7,8]. Other established alternative methods, such as Advanced Oxidation Processes (AOP) [9], have been reported to be effective for the degradation of soluble organic contaminants from water, providing almost total degradation. Several technologies are included under AOP such as photo-Fenton, ozonation, photocatalysis, etc. [10,11].

Thus, the remediation of wastewater with chlorophenols using photocatalysis has been widely studied, but not in the case of 4-CP; however, the complexity of degradation processes with heterogeneous catalysts and the high sensitivity of the reaction to experimental conditions represent two main disadvantages, that make it difficult to compare different related systems. Several studies have evaluated the effects of different substituent groups in the aromatic ring in phenolic compounds, as well as the influence of metal ions and/or oxidant compounds, on the adsorption equilibrium and kinetic parameters of degradation during UV irradiation of a suspension in a photocatalytic process with a semiconductor.

The photocatalytic activity is governed by the catalyst, which is a semiconductor (e.g., TiO_2_ (the most used), ZnO and Fe_2_O_3_) [12]. These compounds have two primary characteristics: the first, their band edge energy; semiconductors with more negative band edge energy than the reduction potential of water (or protons), and that remain stable when are in contact with water can be considered as appropriate catalyst materials [13]; the second characteristic is the particle size, of primary importance in heterogeneous photocatalysis because it is directly related to the efficiency of the catalyst through the definition of its specific surface area [12].

An increasing amount of works has been addressed in searching alternative materials to TiO_2_. Anyway, the information gathered indicates that exist a valid alternative to the use of modified-TiO_2_ for photocatalytic process, although the intrinsic electronic and physico-chemical properties of some compounds reported in the literature suggest more investigation. In the degradation of 4-CP, the TiO_2_ has been applied in the following cases: 1) Photocatalytic degradation method using 1% silver loaded TiO_2_ (Ag-TiO_2_), the degradation/transformation of 4-CP is extremely high compared to neat TiO_2_, this latter requires longer exposure time to obtain the same degree of degradation than the compound Ag-TiO_2_ [14]; 2) Cobalt-modified TiO_2_ (Co-TiO_2_) is able to bring about the photocatalytic degradation of pollutants such as 4-chlorophenol, the best photoactivity was observed for an amount of cobalt between 0.2% and 0.5% Co/TiO_2_ w/w [15]; 3) the mesoporous anatase is able to degrade 100% 4-CP, while Degussa P-25 degraded 57% in time of 180 min, the enhanced photocatalytic activity of the mesoporous titania samples when compared to Degussa P-25 was related to smaller crystallite size, presence of pure anatase phase, higher average pore diameter, and surface area [16]; these are some published works, but in the case of research applying graphite oxide and graphene oxide without doping to degrade photocatalytically 4-CP, it is non-existent until this time.

In the development of new photocatalysts, the graphite oxide (GO), a polymer-like semiconductor made of only carbon, oxygen and hydrogen, has a large exposed area and can be extensively dispersed in water on the molecular scale. These structural features, both due to their chemical and physical aspects, suggest the favourable role of graphite oxide as a photocatalyst. Therefore, graphene oxide (GEO) derivatives of GO can also be good photocatalysts. The difference between them is the number of stacked layers. Both materials (GO and GEO) have photocatalytic activity, as it was reported recently and showed by water splitting and hydrogen production [12,17,18].

Also, nanostructured semiconductor materials are anticipated as new photocatalysts to open up new opportunities and employ the renewable energy sources, such as: Iodine-doped TiO_2_ nanoparticles, titanium oxynitride porous thin films, TiO_2_/SnO_2_ nanofibers, square Bi_2_WO_6_ nanoplates, and Si nanowires. All these materials have been explored and exhibited interesting photoactivities. These materials with optical gap of 2.7 eV have been recognized as a very promising metal free photocatalyst, and its photocatalytic activity is confirmed to be even higher than the commercial nitrogen-doped TiO_2._ But like the TiO_2_, the rapid recombination of electrons and holes is one of the main reasons for the low photocatalytic efficiency of these kinds of photocatalysts [19-21]. But in the case of GO and GEO, the anti-recombination is a given characteristic in current works of research related to the topic of photocatalysts. This effect can be attributed to their nanometric thickness, surface area and the presence of oxygenated groups. Also, both carbon structures can be doped with different materials or atoms to improve their performance as photocatalysts [22]. Additionally, reduced graphene oxide (RGO) modified with different compounds has been used successfully as a photocatalytic material in the remediation treatment of heavy metals and organic compounds [23-26]. Also, a recent work has studied about the photocatalytic effectivity of graphene oxide in the removal of 4-CP by computational method [27]. However, the photocatalytic activities of unmodified GO and GEO have not been studied experimentally, for the removal of organic compounds in water. Thus, it is important to verify the photocatalytic activity of these unmodified carbon materials for the removal of organic compounds in water. GEO has attracted great interest because of its easy availability in bulk quantities, readiness for functionalization by chemical reaction, good dispersion in water and high biocompatibility [26]. Thus, in this research is showed for the first time the capacity of graphene and graphite oxides to remove 4-CP from aqueous solutions under UV-light. Both undoped oxides (GEO and GO) present sufficient activity to remove the organic pollutant from water.

## Materials and methods

### Materials

Graphite (Electron Microscopy Sciences, No. 70230), distilled water (J.T. Baker), 4-chlorophenol (≥99% Sigma Aldrich), Sulfuric acid (H_2_SO_4_, Baker, 98%), potassium permanganate (KMnO_4_, Merck), hydrogen peroxide (H_2_O_2_, Baker, 30%) and distilled water (H_2_O) were used as received. Graphite oxide (GO) and graphene oxide (GEO) were synthesized from graphite by a methodology reported in a previous work [28], and described briefly below.

### Synthesis of graphite and graphene oxides

First, H_2_SO_4_ (46 mL) was added into the reaction flask maintained at 0°C (±2°C) (ice bath), then graphite (2 g) and KMnO_4_ (6 g) were added slowly. After an increase in temperature to 35°C (±2°C), the mixture was stirred by a magnetic stirring bar and mixed for 2 h. Later, the excess water was incorporated to the mixture and H_2_O_2_ (10 ml) was added until there was no gas production.

Then, filtration was performed with distilled water in a glass filter, and the obtained brown GO was dried in an oven (Barnstead Thermoline, Model 3478) at 65°C for 12 h. After that, a solution containing 100 mg of dried GO in 10 mL of H_2_O was prepared. This solution was sonicated for 3 h at room temperature with the assistance of an ultrasonic bath (Branson 1510R-MTH) at 55 degassing units, in order to obtain graphene oxide sheets (GEO).

### Adsorption test

Adsorption test consisted on placing an aqueous solution of 4-CP with a concentration of 30 ppm and neutral pH into a tubular glass reactor, adding graphite oxide or graphene oxide (0.8 g/L in both cases) with magnetic stirring (1000 rpm) and then, the reactor was covered with aluminium foil to prevent contact with external light. The reaction time was 100 minutes and aliquots were taken at 20, 40, 60, 80 and 100 minutes.

### Photolysis test

For these experiments, 30 mL of 4-CP solution (30 ppm) at neutral pH were placed in the same reactor used for the adsorption test and induced with UV irradiation from a lamp (Pencil UV lamp, 254 nm, 5.5 W). Light source was located in the centre of the vessel along with a magnetic stirrer (1000 rpm). The reactor temperature was maintained at 24°C +/- 2°C and the reactor was covered with aluminium foil to prevent contact with external light. The reaction time was 100 minutes and aliquots were taken after 20, 40, 60, 80 and 100 minutes.

### Photocatalyst test

The photocatalytic efficiencies of the GO and GEO were determined in the same tubular glass reactor used for the photolysis test with the same continuous stirring (1000 rpm). The total reaction volume was 30 ml. The tests were performed using 0.8 g/L of graphite and graphene oxides, with 30 ppm of 4-CP at neutral pH. The reactor temperature was maintained at 24°C +/- 2°C and aluminium foil was placed around the reactor. A UV lamp (Pencil UV lamp, 254 nm, 5.5 W) was placed in the centre of the reactor to provide UV light radiation. The reaction time was 100 minutes and aliquots were taken after 20, 40, 60, 80 and 100 minutes.

### Chemical Oxygen Demand (COD)

Method 8000/reactor digestion method in the high range (0–1500 mg/L), programmed in the HACH DR5000 spectrophotometer was used to determine the COD in the initial and final solutions of 4-CP.

### Characterization

Fourier transform infrared spectroscopy (FT-IR) of GO and GEO samples was performed using a Bruker-Vector 33 with a scanning range of 4000-500 cm^−1^ with resolution of 1 cm^−1^.

Raman spectroscopy of the carbon samples was carried on a Micro-Raman (Dilor, Lab Ram), with measurements at 488 nm incident laser light with a spectral resolution of 1 cm^−1^.

UV-vis spectroscopy was carried out on a HACH DR5000 spectrophotometer at wavelengths of 200–1100 nm to determine the absorption bands characteristic of the 4-CP solution and monitor the progress of 4-CP removal from the solution by adsorption, photolysis and the photocatalytic processes.

HPLC (High Performance Liquid Chromatography) was performed with a mobile phase of H_2_O_2_ with 5 mmol H_2_SO_4_ and methanol (80:20), a flow rate of 1 mL/min and an Ascentis Express C_18_ 3 cm × 4.6 mm (2.7 μm) SUPELCO column for the determination of intermediate compounds.

Mineralization of 4-CP was followed by measuring the chemical oxygen demand (COD) in a typical reaction. An aliquot was taken at the end of the reaction and the COD was measured by a colorimetric method on the HACH DR5000 spectrophotometer.

## Results and discussion

### FT-IR spectroscopy

**Figure 1:**
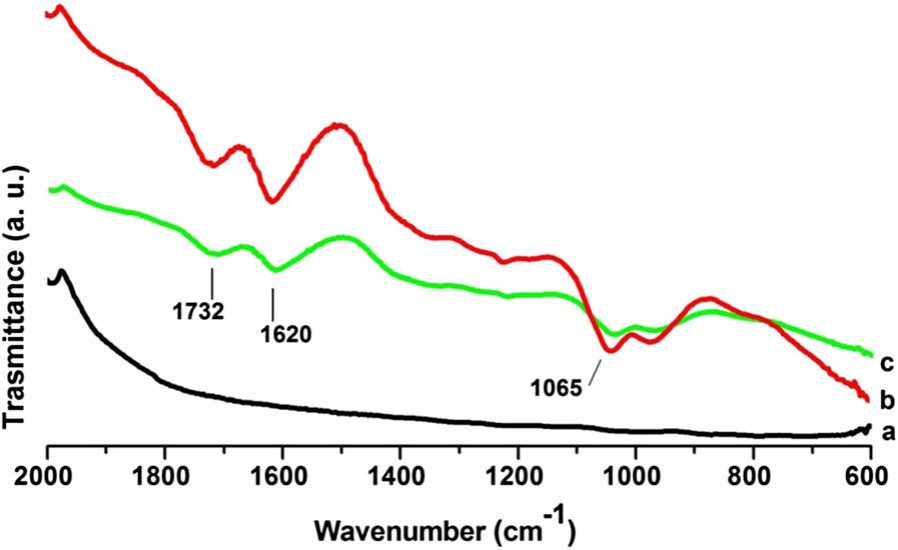
FT-IR spectra of graphite (a), (b), (c).

The effect of chemical modification due to reaction of graphite with acids is observed in the FT-IR spectra (Figure 1). Pure graphite is inactive as is observed in spectrum (a), whereas GO and GEO spectra (b and c respectively) show several distinctive signals such as: 1732 cm^−1^, v(C = O) at carboxyl groups; 1620 cm^−1^, v(C = C); and 1065 cm^−1^, v(C-O), giving evidence for the presence of oxygen-containing groups, caused by the chemical reaction and reported in previous work [28].

### Raman spectroscopy

**Figure 2:**
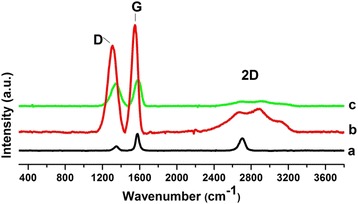
Raman spectra of: graphite (a), graphite oxide (b), graphene oxide (c).

Raman spectroscopy shows the characteristic bands assigned to the carbon materials, indicating the modification in the hybridization and defects produced at the surface of materials synthetized by chemical reaction. Figure 2, shows distinctive signals: a strong band close to 1580 cm^−1^ (G band), a line around 1350 cm^−1^ (D band) and 2D band in the region of 2700 cm^−1^, characteristic for GO and GEO, these bands have been reported by different authors and also in a previous work of this research group [28].

### UV-vis spectroscopy

**Figure 3:**
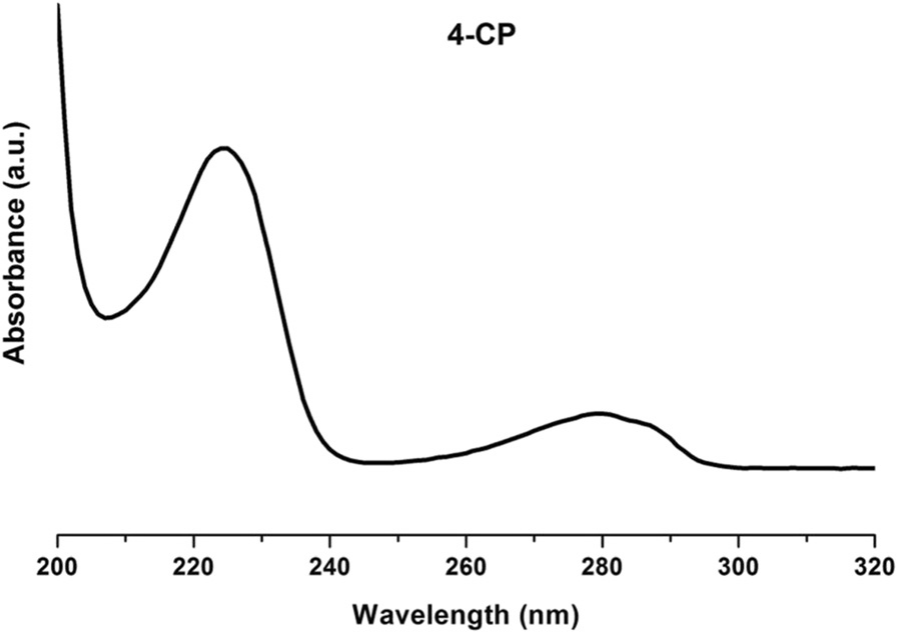
UV-Vis spectrum of 4-chlorophenol (4-CP).

Figure 3 shows the UV-vis spectrum of 4-CP; two characteristic bands of this compound as aqueous solution are observed at 280 and 225 nm [3,29].

**Figure 4:**
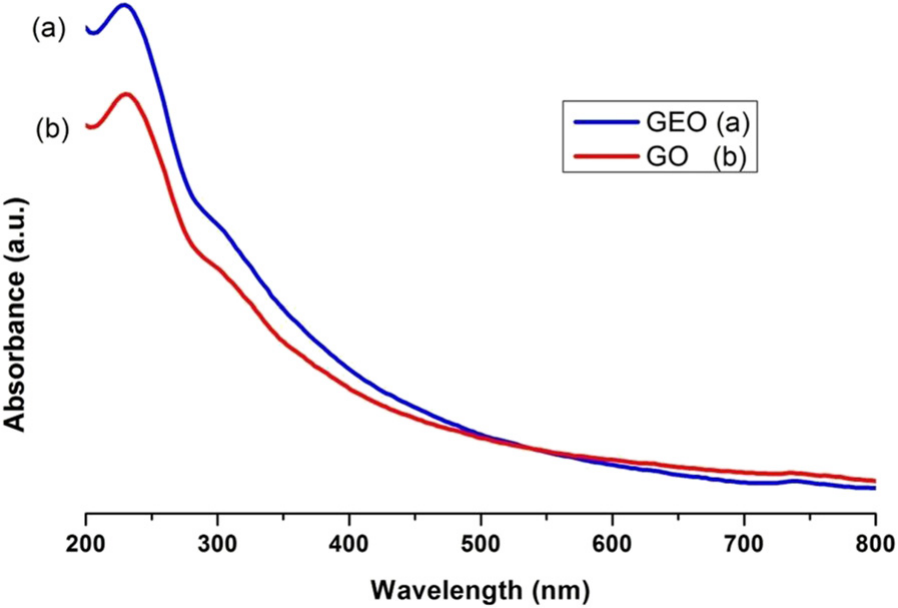
UV-Vis spectra of graphene oxide-GEO (a) and Graphite Oxide-GO (b).

The UV-vis spectra in Figure 4 correspond to GO and GEO and show the maximum absorption peaks at 227 and 305 nm that each material exhibits, the bands are attributed to the π-π* electronic transitions of the aromatic C-C bonds and the n-π* transition of the C = O bond, respectively [25]. These spectra are also used to obtain the band gap values for both oxides. Tauc’s method is applied, and the calculated values are around 2–4.7 eV for graphite oxide and 1.8–4 eV for graphene oxide; these values are comparable with other semiconductors used as photocatalysts; in addition, these results are very similar to those reported in previous work [13].

### Adsorption test

**Figure 5:**
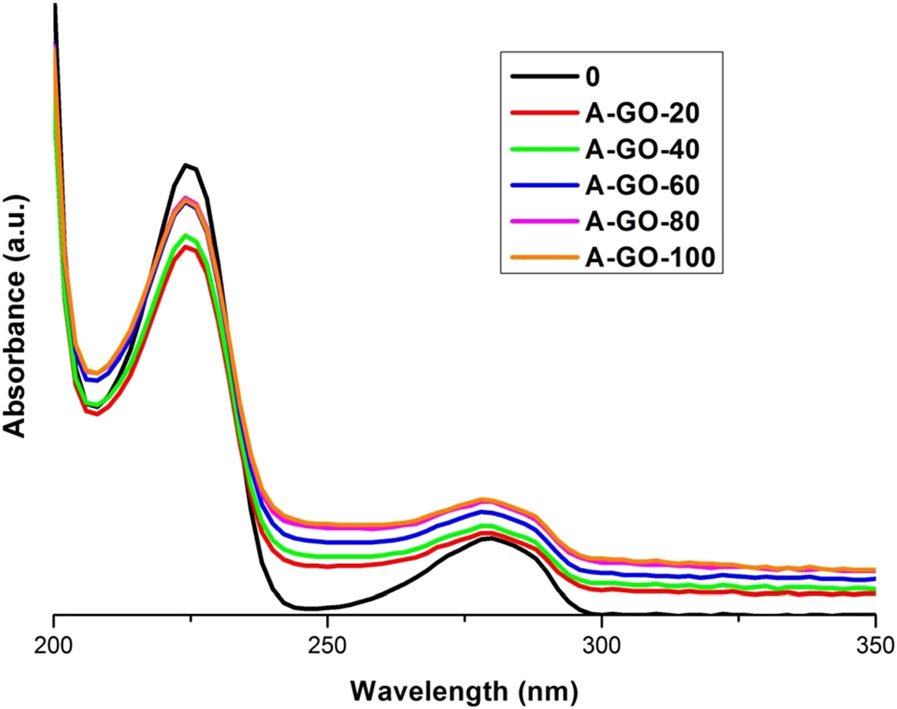
UV-Vis spectra of adsorption test for 4-CP solution using GO as adsorbent. Taking aliquots at 0, 20, 40, 60, 80 and 100 minutes of reaction.

**Figure 6:**
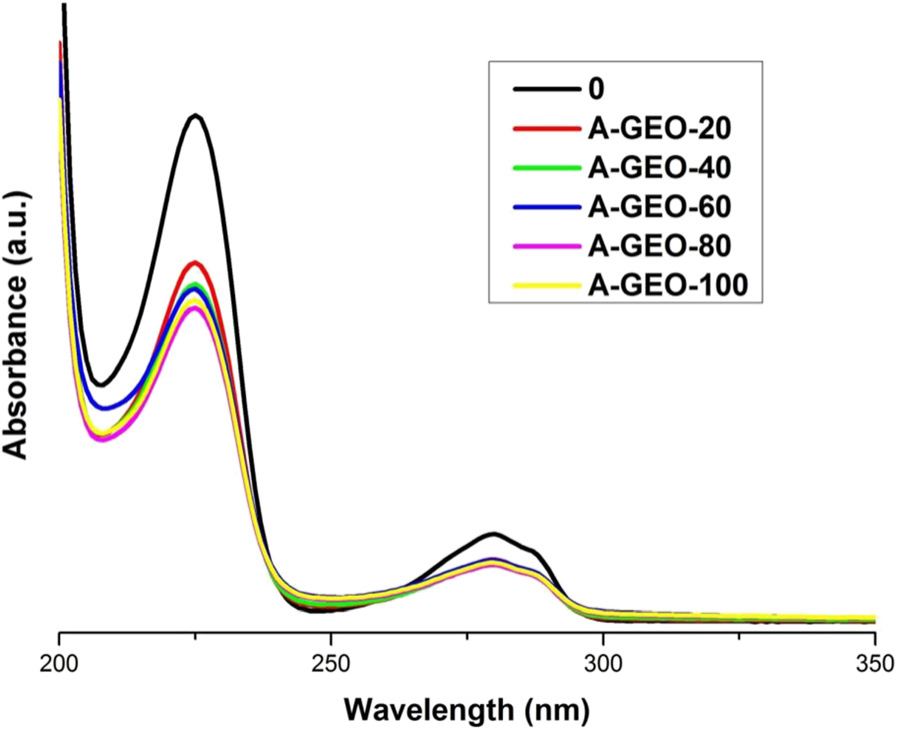
UV-Vis spectra of adsorption test for 4-CP solution using GEO as adsorbent. Taking aliquots at 0, 20, 40, 60, 80 and 100 minutes of reaction.

To confirm the degradation of 4-CP due to photocatalysis, samples of graphite and graphene oxides are tested as adsorbents of this pollutant. Figure 5 shows 4-CP removal using GO (A-GO) as adsorbent. In this process, it is observed that the absorption intensity of the band at 280 nm produced by the solution after adsorption with GO is more intense in comparison with the band of only 4-CP (sample 0), indicating that under these conditions GO adsorbs molecules of 4-CP; however, the solution shows a light yellow colour, indicating that the adsorption process may produce some by-products, since it cannot degrade the benzene ring of the 4-CP. The interaction between the two compounds in water may lead to derivatives and by-products of 4-CP, corroborated by the yellow colour of the solution and the increment in absorption of the corresponding band at 225 nm for the benzene ring. Thus, this effect indicates that 4-CP is being adsorbing via link in the GO by C–Cl linkages, with 29% of the 4-CP ultimately removed (calculated by calibration curves used in each experiment). In the case of GEO in the adsorption test (A-GEO), Figure 6 shows the spectra of the carbon samples, where the band at 280 nm decreases; although, in the same way, it can be seen that the band is not eliminated, this could indicate that the functional groups present in the graphene oxide adsorb the complete molecule in a different way than GO. GEO is more efficient in adsorption and it can be considered that no intermediate groups are generated. In the case of GO, the oxygen-containing functional groups between layers do not interact with the 4-CP. However, in 2 dimensions GEO produces better adsorption. The band at 225 nm in the GEO absorption spectra decreases more than in the GO spectra. GEO has a higher capacity as an adsorbent material, with 36% of the removed pollutant (4-CP). These adsorption results are useful when comparing the performance of GO and GEO as photocatalytic materials. Those tests are presented in the next sections.

### Photolysis test

Photo dissociation of the C-Cl bond in 4-CP has been reported using UV laser excitation [30]. The photochemical reaction is the first stage in the C-Cl bond cleavage of 4-CP, with subsequent decomposition of the intermediate ion or radical [31]. It is important to consider the band at 280 nm, which defines the generation of intermediate compounds, in the progression of 4-chlorophenol degradation.

**Figure 7:**
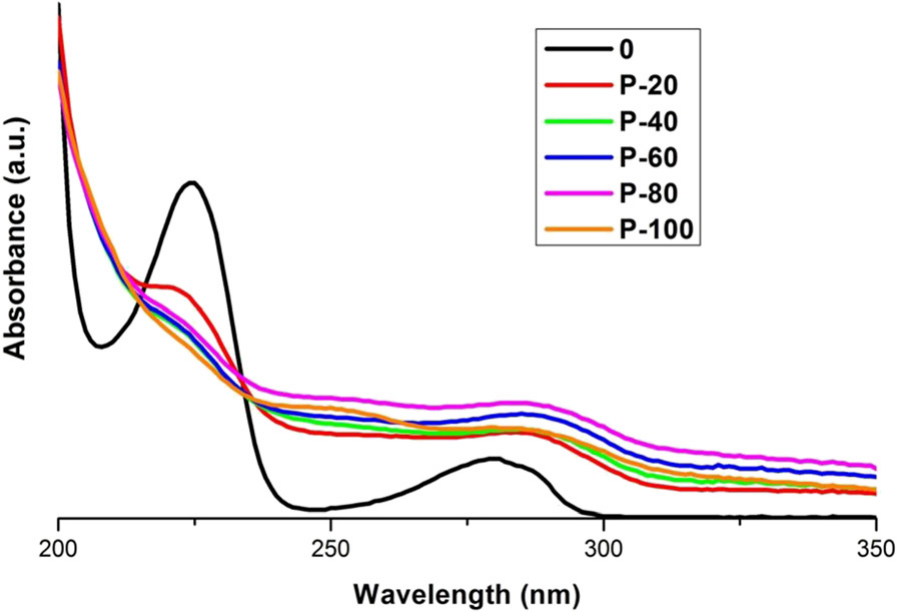
UV-Vis spectra of photolysis test for 4-CP solution. Taking aliquots at 0, 20, 40, 60, 80 and 100 minutes of reaction.

Figure 7 shows UV-vis spectra obtained from the photolysis test, which corroborate the interaction between the UV light and the water. It is observed, that the characteristic bands in the UV-vis spectrum of 4-CP are affected, and degradation is evident. This indicates that the wavelength emitted by the used lamp is capable of activate OH groups in the solution, causing oxidation due to air trapped in the reactor where the reaction takes place and breaking the C-Cl bonds present in 4-CP. Also, as presumed, the generation of intermediate compounds is apparent, because in the spectrum obtained we can see that the band at 280 nm has a significant shift and increased absorption. It is important to remember, that the by-products in many cases are more toxic, so that the photolysis process alone is not a sufficient method to completely degrade 4-CP.

### Photocatalytic activity

**Figure 8:**
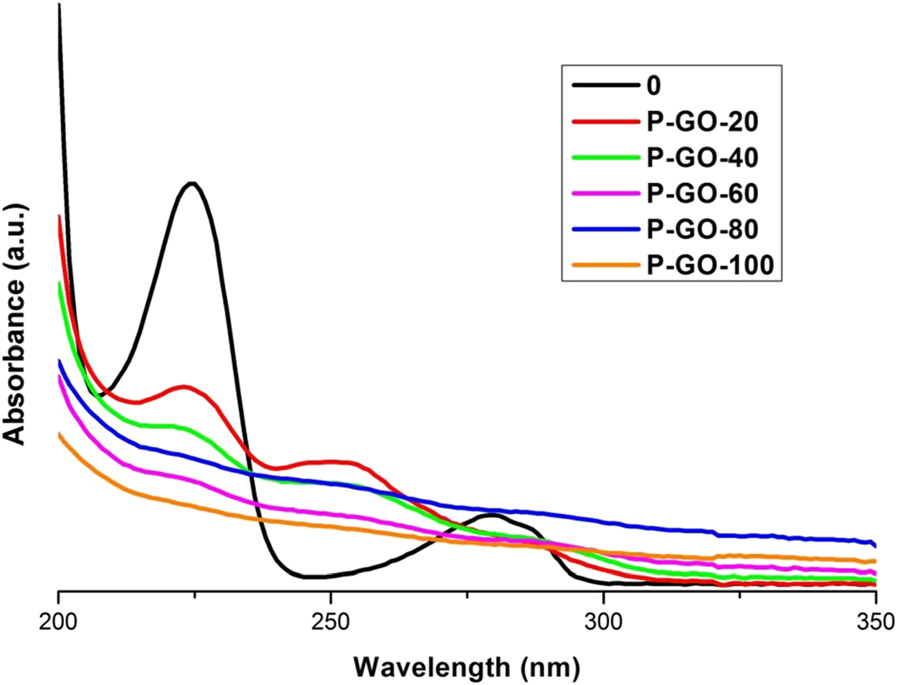
UV-Vis spectra of photocatalytic test for 4-CP solution using GO as photocatalyst. Taking aliquots at 0, 20, 40, 60, 80 and 100 minutes of reaction.

**Figure 9:**
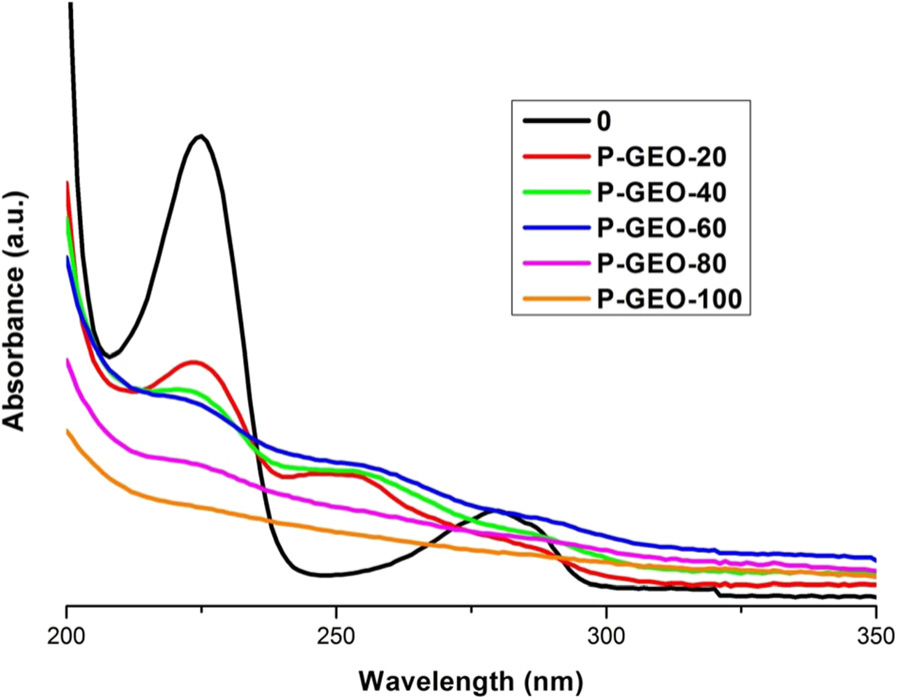
UV-Vis spectra of photolysis test for 4-CP solution using GEO as photocatalyst. Taking aliquots at 0, 20, 40, 60, 80 and 100 minutes of reaction.

Figures 8 and 9 show the UV-vis spectroscopy obtained during the photocatalytic tests using GO (P-GO) and GEO (P-GEO) as photocatalysts, respectively. The degradation of the absorption bands of 4-CP are clearly observed. Comparing the absorption spectra of the two photocatalysts, it can be observed that the degradation of 4-CP is very similar, but after 20 minutes of photocatalysis reaction, the band at 225 nm diminish more considerably with GEO (Figure 9) than with GO (Figure 8). This effect, observed in both spectra, implies that in the GEO the energy caused by UV light at 254 nm is able to quickly activate the movement of electrons, generating OH radicals in the carbon surface, allowing better contaminant degradation. However, in GO, this effect is less significant. It is suggested, that the size (carbon dimension) and disposition of functional groups play an important role in 4-CP degradation. Also, the spectra of Figures 8 and 9 show that the band at 280 nm disappears from all the samples. This effect is attributed to a minimal generation of intermediate compounds, a good photocatalytic response and the degradation of the pollutant by both materials (GEO, GO). In addition, these results showed that degradation obtained via photocatalysis using GEO is faster than that achieved with GO.

**Figure 10:**
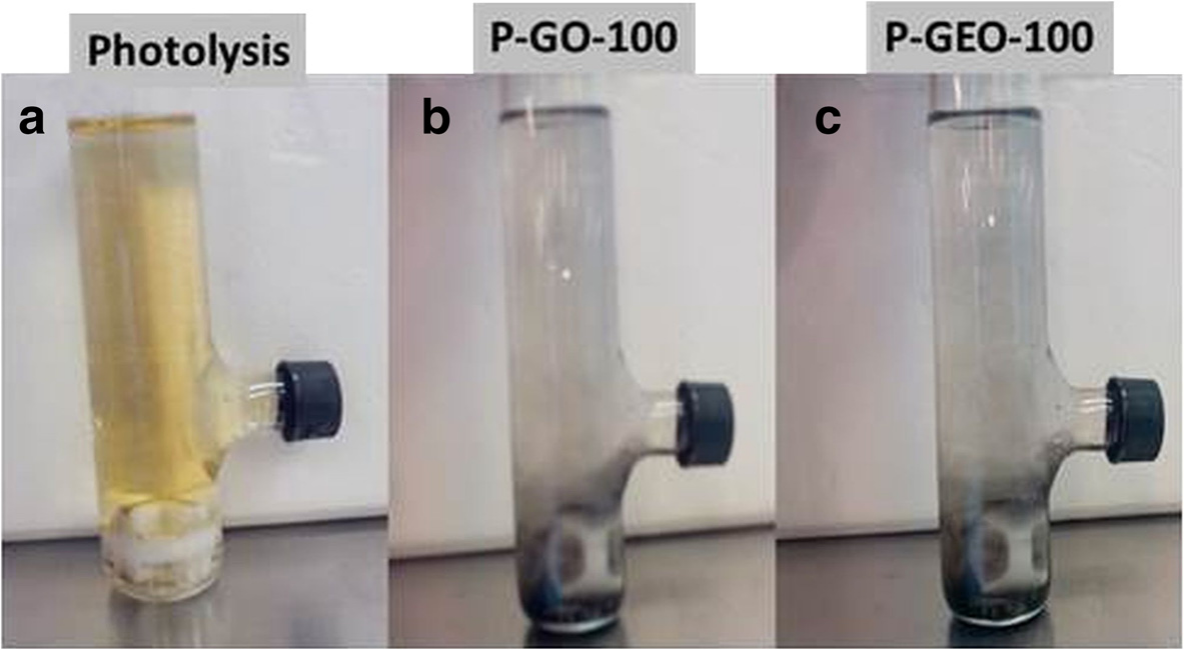
Pictures of the final solutions from the Photolysis. **(a)**, Photocatalytic process with graphite oxide (P-GO) **(b)** and Photocatalytic process with graphene oxide (P-GEO) **(c)**. Differences in colour between yellow and colourless is observed, indicating the generation of by-products (yellow solution).

As it was mentioned before, a common characteristic of the presence of by-products generated by chlorophenols, as result of degradation process, is the yellow colouration of the solution, which indicates that intermediates are generated; however, if the final solution is transparent, it indicates that by-products generated are not excessive. Compounds such as hydroquinone and benzoquinone give a yellow colour to the final solution of a treated chlorophenol. Figure 10 shows the final solutions obtained by photolysis (Figure 10a) and photocatalysis with GO (Figure 10b) and GEO (Figure 10c), respectively, it is observed that the yellow colour is prominent after photolysis, but the solution is only slightly yellow after photocatalysis performed with GO and practically colourless in photocatalysis with GEO, indicating that the best process for removing 4-CP is photocatalysis with GEO.

**Figure 11:**
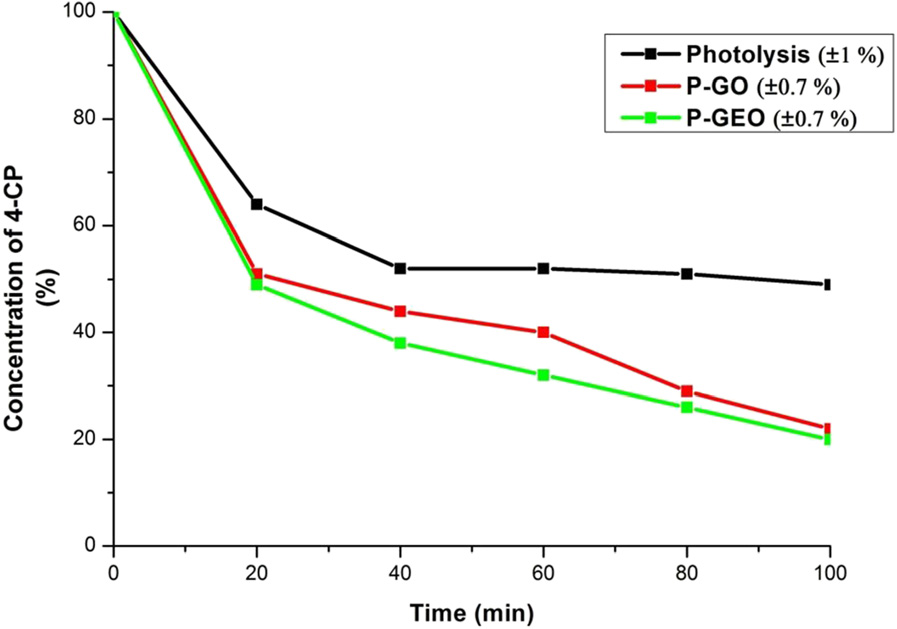
Degradation percentage (%) of concentration of 4-CP solution in the photolysis. Photocatalytic process with graphite oxide (P-GO), and Photocatalytic process with graphene oxide (P-GEO), at 100 min of reaction time. Values of percentage error are included in parentheses.

On the other hand, in Figure 11 is observed over 50% of 4-CP degradation at 20 minutes by the sample of GEO; with photocatalysis using GO, the percentage of 4-CP removed is very similar, indicating that both photocatalysis tests have superior removal compared to the photolysis process. Photolysis at 100 minutes reaches 50% of 4-CP degradation in 100 minutes, while GO and GEO achieve 78% and 81% of removal in the photocatalysis test, respectively. The characteristic bands (225 nm and 280 nm) disappear independently in the spectra of GO and GEO in the photocatalyst tests, it is standard to calculate the removal percentage with the band at 225 nm, which is the distinguishing band for this quantification [29-32]. Also in the Figure 11, it can be observed that the degradation process is faster in photocatalysis with GEO than using the other two processes. This can be attributed to structural features of the GEO that have already been mentioned in this paper, as well as in other researches [13,17,18,20,33]; thus, it is presumed that the oxygen-containing functional groups present in the GEO sheets function as oxidizing radicals in the process and accelerate the degradation of 4-CP, influenced by the nanometric size of GEO and carbon material in 2 dimension. The enhanced photocatalytic activity for GEO could be attributed to the ability to capture and transport electrons, and to promote charge separation. It is known that the higher separation efficiency of electron–hole pairs will enhance photocatalytic activity and results in a large number of holes participating in the photocatalytic process [34]. On the other hand, it is important to note that electron-hole recombination could be lower in GEO compared to GO. However, again, the advantages of corrugated sheets of GEO favour the available surface area; this is the great advantage of this graphitic material as a photocatalyst compared to conventional photocatalysts.

Degradation of 4-CP, using TiO_2_ as photocatalysts activated in UV zone supported in different materials such as: activated carbon, silica, zeolite have been reported [35,36]. Also graphene has been used as supported of other materials to produce photocatalytic effect in other researches [37]. However, carbon materials without doping, or specifically graphene oxide materials as photocatalysts have not been reported to degrade 4-CP. Other materials activated in UV zone used as photocatalysts in the degradation of 4-CP are enlisted in the Table 1 in comparison with the carbon materials (GO and GEO) analysed in this research. It is observed that GO and GEO are efficient materials for the degradation of this organic molecule in comparison with other photocatalysts, either compounds or hybrid materials.

**Table 1:** **Materials used as photocatalyst to removal 4-CP at different reaction times**

**Catalysts**	**Removal of 4-CP (%)**	**Reaction time (min)**	**Reference**
**Ag–TiO2**	98	60	[14]
**TP2.5 (P-modified TiO2)**	78	270	[32]
**TiO2-325 mesh**	97	180	[35]
**MgO TiO2**	100	120	[38]
**An(P25):Ru(P25)**	80	120	[39]
**Mesoporuos titania**	77-100	180	[16]
**ZnO**	75	75	[40]
**TiO2 synthesized**	84	75	[40]
**ZnO-TiO2**	93	75	[40]
**Graphite oxide (GO)**	92	100	*This research*
**Graphene oxide (GEO)**	97	100	*This research*

### Intermediate degradation of 4-chlorophenol analysed by HPLC

Table 2 shows the concentrations of by-products generated in the reactions of photolysis and photocatalysis as determined by HPLC. In the photocatalytic degradation with both catalysts and with photolysis, aromatic intermediates and carboxylic acids are detected. Three aromatic intermediates are generated during 4-CP degradation, namely benzoquinone, 4-chlorocatechol and hydroquinone. It is known that these three aromatic compounds are commonly the intermediates generated in the TiO_2_ photocatalytic degradation of 4-chlorophenol [14,31]. The HPLC analysis was performed on the final sample from each reaction, after 100 min of photolysis or photocatalysis with GO and GEO. Considering the initial 4-CP concentration of 30 ppm, all three processes gave successful degradation. The concentration of benzoquinone in the three cases is the same, 4.8 ppm. The concentration of 4-chlorocatechol is very low, since it is determined to be less than 1 ppm in the final solution in the case of photocatalysis and above 1 ppm in photolysis, although the difference of 0.3 ppm reflects the more effectiveness of the photocatalytic process employed. In the Table 3 it can be observed that the intermediate hydroquinone is present in the samples treated with photolysis and GO as photocatalyst; however, the sample treated with GEO as a photocatalyst does not contain this intermediate. These results indicate that the degradation of 4-CP is more effective with photocatalysis, and GEO is more effective in the degradation process.

**Table 2:** **Concentration in ppm of aromatic intermediate compounds generated during the degradation of a 4-CP solution in photolysis and photocatalysis**

**HPLC**	**Aromatics compounds (ppm)**		
	**Benzoquinone**	**4-chlorocatechol**	**4-chlorophenol**
**Photolysis**	4.8	1.1	0.7
**P-GO**	4.8	0.8	0.4
**P-GEO**	4.8	0.8	0.2

**Table 3:** **Concentration in ppm of intermediate compounds (carboxylic acids) generated during the degradation of a 4-CP solution by photolysis and photocatalysis**

**HPLC**	**Carboxylic acids (ppm)**			
	**Oxalic acid**	**Formic acid**	**Succinic acid**	**Hydroquinone**
**Photolysis**	5.1	2.4	3.3	2.7
**P-GO**	3.8	0.5	2.4	1
**P-GEO**	3.3	0.5	1.5	0

Also, in Table 3, the carboxylic acids generated in the 4-CP degradation process are showed. The highest concentration obtained is oxalic acid, at 5.1 ppm after photolysis; however, in the photocatalytic reactions with GO and GEO the concentration of oxalic acid is 3.8 and 3.3 ppm, respectively. Moreover, in the production of succinic acid the lowest concentration is present in the solution from the photocatalytic process with GEO. The by-products generated in our experimental processes are very similar with those found in other reports, although the amounts or concentrations of these compounds are not reported previously [29,38].

### Mineralization of 4-chlorophenol evaluated by COD

**Figure 12:**
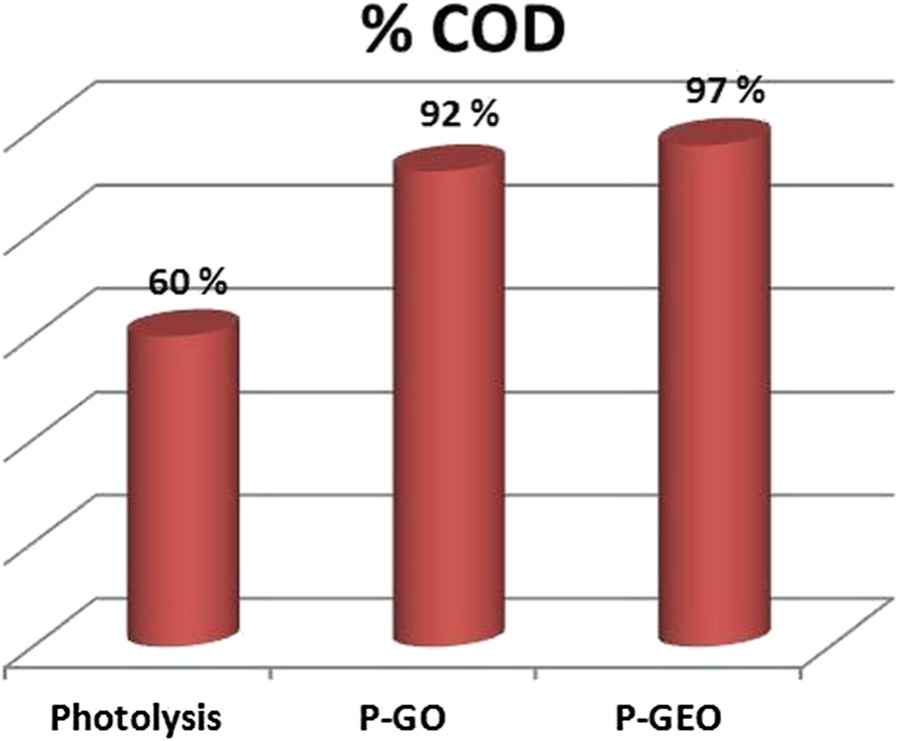
Degradation percentage (%) of organic matter (COD). Determined at the end of the photolysis and photocatalysis tests with GO and GEO (P-GO and P-GEO) in the degradation of 4-CP.

The COD test is used to measure the total quantity of oxygen-consuming substances during the complete chemical breakdown of organic substances in water. Figure 12 illustrates the mineralization of 4-CP percentages achieved by photolysis, and photocatalysis with P-GO and P-GEO. It is possible to observe that, the photolysis process eliminates 60% of organic matter, but the photocatalytic processes remove more than 90% (92% and 97% for P-GO and P-GEO, respectively). In addition it is worthy of indicate that the by-products generated in the photocatalytic reaction processes are practically eliminated, in contrast with the photolysis process, because the intermediate compounds are present at the same time in the reactions. Thus, the fact that the photolysis process alone is not enough to degrade 4-CP and the intermediate compounds is corroborated.

## Conclusions

According with the results showed in this research, it can be concluded that GO and GEO are materials with sufficient capacity to degrade 4-CP present in water as contaminant. This fact promises a new trend in the application of carbon nanomaterials without doping as photocatalysts. UV determinations showed that the degradations of 4-CP in 92% and 97% using GO and GEO at 100 min respectively, are superior to photolysis (50%) during the same time, it was found that the reaction mechanism is favourable for 4-CP degradation with carbon oxides. The main by-products generated during the photolysis, and photocatalytic method with GO and GEO, correspond to aromatic compounds and carboxylic acids. In the photocatalytic process the by-products generated were lower in comparison with photolysis process, and better results were obtained with photocatalysis performed with GEO, in this case hydroquinone was not observed at the end of the reaction. Also, mineralization measured by COD indicates that up to 97% of organic matter is removed through photocatalysis using GEO. In addition, research findings show that nanometric size plays an important role in photocatalytic processes, reflected in the fact that GEO show better results in eliminating 4-CP than GO. Materials such as GO and GEO that have recently been used in photocatalytic applications such as water splitting [13,17] are showed in this research as an efficient alternative to eliminate 4-CP in water, using a small quantity of photocatalyst and a short reaction time. Thus, both materials are suggested as effective for their use in advanced oxidation processes. Thus, it can be concluded that, photocatalysis with GO and GEO is simple and effective, because, avoids the use of any oxidizing agent. Furthermore, their lower rates of recombination could be an important factor in this photocatalytic process. Therefore, the highly efficient degradation of 4-CP using GO and GEO as photocatalytic materials could be extended to other organic materials with aromatic rings, and thereby a new line of research in photocatalysis with carbon nanomaterials focused in the treatment of contaminated water, depending on the structural features of these materials, their electronic properties, dimensions and functional groups in the materials’ surface.
